# Deleterious Variants in Intolerant Genes Reveal New Candidates for Self-Limited Delayed Puberty

**DOI:** 10.1093/ejendo/lvaf061

**Published:** 2025-03-27

**Authors:** Raíssa C. Rezende, Wen He, Lena R. Kaisinger, Antonio M. Lerario, Evan C. Schafer, Katherine A. Kentistou, Priscila S. Barroso, Nathalia L. M. Andrade, Naiara C. B. Dantas, Elaine F. Costa, Laurana P. Cellin, Elisangela P. S. Quedas, Stephanie B. Seminara, Rodolfo A. Rey, Romina P. Grinspon, Veronica Meriq, Ken K. Ong, Ana Claudia Latronico, John R. B. Perry, Sasha R. Howard, Yee-Ming Chan, Alexander A. L. Jorge, Additional members of the Delayed Puberty Genetics Consortium are, Leo Dunkel, Aristeides Giannakopoulos, Paulina Merino

**Affiliations:** Centre for Endocrinology, William Harvey Research Institute, Barts and the London School of Medicine and Dentistry, Queen Mary University of London, London UK; University Hospital of Patras, Rio, 26504 Patras Greece; Institute of Maternal and Child Research, Faculty of Medicine, University of Chile, Santiago, Chile; 1 Unidade de Endocrinologia Genetica (LIM 25), Hospital das Clínicas da Faculdade de Medicina, Universidade de São Paulo (USP), São Paulo, SP, 0124690, Brazil; 2 Division of Endocrinology, Department of Pediatrics, Boston Children’s Hospital, 300 Longwood Avenue, Boston, MA 02115, USA; 3 MRC Epidemiology Unit, Institute of Metabolic Science, University of Cambridge School of Clinical Medicine, Cambridge CB2 0QQ, UK; 4 Division of Metabolism, Endocrinology, and Diabetes, Department of Internal Medicine, University of Michigan, Ann Arbor, MI 48109, USA; 5 Massachusetts General Hospital Harvard Center for Reproductive Medicine and Reproductive Endocrine Unit, Massachusetts General Hospital, Bartlett Hall Extension, 5th Floor, 55 Fruit Street, Boston, MA 02114, USA; 6 Centro de Investigaciones Endocrinológicas “Dr. César Bergadá” (CEDIE), CONICET–>FEI–Divisièn de Endocrinología, Hospital de Niños Ricardo Gutiérrez, Gallo 1330, C1425EFD Buenos Aires, Argentina; 7 Institute of Maternal and Child Research, Faculty of Medicine, University of Chile, Santa Rosa 1234, 2° piso, Santiago 8320000, Chile; 8 Metabolic Research Laboratory, Institute of Metabolic Science, University of Cambridge School of Clinical Medicine, Cambridge CB2 0QQ, UK; 9 Centre for Endocrinology, William Harvey Research Institute, Queen Mary University of London, Charterhouse Square, London EC1M 6BQ, UK; 10 Department of Pediatrics, Harvard Medical School, 25 Shattuck Street, Boston, MA 02115, USA

**Keywords:** Self-limited delayed puberty, Delayed puberty, Exome sequencing, Genetic association studies

## Abstract

**Objective:**

Self-limited delayed puberty (SLDP) is the most common cause of delayed puberty and exhibits high heritability, although few causal genes have been identified. This study aims to identify potential candidate genes associated with SLDP.

**Methods:**

Whole-exome sequencing was conducted in 71 children with SLDP, most of whom presented with short stature. Rare coding variants were prioritized through comprehensive bioinformatics analyses and classified as high-impact or moderate-impact based on predicted functional effects. Candidate genes were selected based on the absence of human phenotype data, recurrence within the cohort, intolerance to mutation, and prior identification in genome-wide association studies. Burden tests compared the frequency of rare high-impact variants in these candidate genes between SLDP patients and the gnomAD v2.0 control group. Gene-phenotype associations were further explored using UK Biobank data.

**Results:**

Fourteen high-impact and seven moderate-impact variants were identified in 19 candidate genes, suggesting a potential role in SLDP. Variants in eight candidate genes (*GPS1, INHBB, SP3, NAMPT, ARID3B, NASP, FNBP1, PRDM2*) were significantly enriched in cases compared to controls in the burden test analysis. *INHBB* was additionally linked to delayed menarche in UK Biobank data. Furthermore, three pathogenic variants (*CDK13, GDF5, ANRKD11*) and six likely pathogenic variants (*TYMP, DPF2, KMT2C, TP63, MC3R, GHSR*) previously associated with growth or pubertal human disorders were identified.

**Conclusion:**

These findings suggest that SLDP involves both monogenic and polygenic mechanisms, with novel candidate genes contributing to its genetic basis. The association of *INHBB* with pubertal timing underscores its potential role in SLDP pathophysiology.

## Introduction

Puberty is a fundamental aspect of human development, marking the transition from childhood to adulthood through sexual maturation and growth acceleration^1^. The age at which puberty begins follows a roughly normal distribution within the population, with the typical onset ranging from approximately 8 to 13 years for girls and 9 to 14 years for boys. The appearance of secondary sexual characteristics before or after these age ranges is classified as precocious or delayed, respectively^2^.

Delayed puberty can result from various conditions, such as self-limited delayed puberty (SLDP), transient or permanent hypogonadotropic hypogonadism, and hypergonadotropic hypogonadism^3^. Although these conditions may present similarly in their early stages, their therapeutic approaches differ from clinical monitoring to continuous hormone replacement^3^. Therefore, distinguishing the underlying etiological mechanisms of pubertal delay is essential for appropriate medical management.

In addition to facilitating reproductive capacity, the increased production of sex hormones during puberty amplifies growth hormone (GH) signaling pathways, contributing to the growth acceleration^1^. The absence of this effect is evident in many children with SLDP, who often present with delayed bone maturation, a slow growth rate due to delayed growth spurt, and, in some cases, short stature^3^. Notably, not all children with SLDP exhibit short stature, nor will they necessarily remain short in adulthood.

Genetics plays a pivotal role in determining the age of puberty onset. Previous studies estimated that hereditary factors account for 50-80% of its variability^4–6^. Genome-wide association studies (GWAS) have identified numerous small-effect genetic signals that influence the timing of puberty^7,8^. Additionally, certain cases of precocious and delayed puberty have been linked to defects in single-gene models^9–16^. Particularly, the primary causes of delayed puberty—SLDP and hypogonadotropic hypogonadism —exhibit a strong genetic component. In individuals with congenital hypogonadotropic hypogonadism, a causal genetic variant can be identified in up to half of the cases^17^. In contrast, while a positive family history of pubertal delay is described in most individuals with SLDP, the number of implicated genes is limited, and a definitive genetic diagnosis is infrequent ^12–16,18,19^.

The genetic basis of SLDP is intricate and influenced by multiple factors. As an extreme manifestation of a quantitative trait affecting approximately 2% of the population, potential causal genetic variants may be present in population databases but overlooked due to filtering criteria that prioritize rare variants. The inheritance patterns of SLDP are diverse, possibly encompassing both monogenic and polygenic mechanisms. Additionally, environmental factors can further influence the timing of puberty onset through epigenetic modifications.

Employing a "hypothesis-free" approach through whole-exome sequencing (WES) may facilitate the identification of new candidate genes for SLDP. Furthermore, there is a paucity of studies specifically investigating children and adolescents with significant short stature associated with pubertal delay. Consequently, our study aimed to identify novel candidate genes in a cohort of patients with SLDP characterized by short stature.

## Methods

### Ethical aspects and patient selection

The present study followed the principles of the Declaration of Helsinki and was approved by the Ethics Committee of Hospital das Clínicas da Universidade de São Paulo (registration number 37868114.3.0000.0068). We obtained formal consent from patients or parents for clinical and genetic analysis. We conducted a cross-sectional observational evaluation of individuals with a confirmed diagnosis of SLDP followed in a tertiary center. The patient selection criteria were previously described^20^. Briefly, pubertal delay was defined as girls with delayed breast development and boys with testicular volume more than 2.25 standard deviations (SD) below the expected for age^21^. When physical examination data were unavailable for boys, serum testosterone values below the 5th percentile for age were considered suggestive of delayed puberty^22,23^. Diagnostic confirmation of SLDP was established by the onset of pubertal development before age 18, either spontaneously or following pubertal induction (Supplemental Figures 1 and 2). Individuals with identifiable underlying causes of short stature and pubertal delay were excluded^24^.

### Data collection and statistical analysis

Anthropometric data were expressed as Z-scores for age and sex^25^. Pubertal development was assessed using Tanner breast stages in girls and testicular volume in boys^26,27^. Height at puberty onset was recorded at Tanner stage 2 in girls and testicular volume ≥4 mL in boys. Final height categories included adult height (no height variation for six months) and nearly adult height (growth velocity <2 cm/year for six months). Family history of short stature was defined as having a parent with a height Z-score ≤ -2 SD, while delayed puberty was defined as menarche ≥15 years in female relatives or delayed secondary sexual development in male relatives. Qualitative data were summarized as counts and percentages, quantitative data as medians and interquartile ranges, and comparisons used Chi-square or Fisher's exact tests.

### Genetic evaluation

DNA was extracted from peripheral blood leukocytes, and WES was conducted at the Broad Institute of MIT and Harvard, Cambridge, Massachusetts, USA. This analysis was conducted as part of the Delayed Puberty Genetics (DPGen) Consortium, which investigates the genetics of pubertal delay. Exome sequencing was performed using Illumina’s Rapid Capture Exome Kit and automated with the Agilent Bravo system. Sequencing was conducted on HiSeq 2000/2500 platforms using v3 chemistry, and data were processed with Freebayes^28^ and annotated by ANNOVAR^29^. The average exome coverage was 121.5x, with 96.9% of target regions covered at >20x depth.

**Figure 1:**
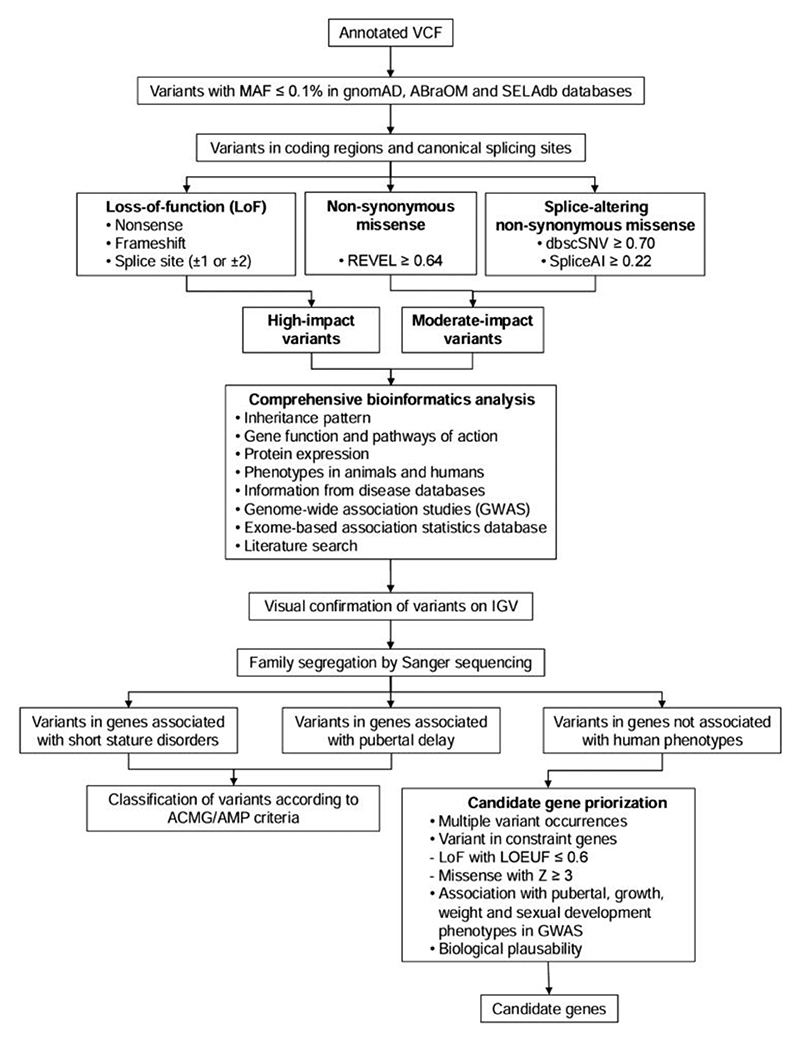
Flowchart for variant prioritization and candidate gene identification VCF: variant call format; MAF: minor allele frequency; gnomAD: Genome Aggregation Database; ABraOM: Brazilian Online Mutation Archive; SELAdb: Large-Scale Sequencing database; REVEL: Rare Exome Variant Ensemble Learner; dbscSNV: Splicing Consensus Single Nucleotide Variant Database; SpliceAI: Artificial Intelligence Splicing; IGV: Integrative Genomics Viewer; ACMG: American College of Medical Genetics and Genomics; AMP: Association for Molecular Pathology; LOEUF: Loss-of-Function Observed/Expected Upper Bound Fraction; GWAS: genome-wide association studies.

Figure 1 describes the variant filtering protocol. Variants with a minor allele frequency (MAF) below 0.1% in population databases (gnomAD^30^, ABraOM^31^, and SELAdb^32^) were selected if they were present in coding regions or canonical splicing sites. We classified variants into two categories: high-impact (i.e., rare, high-confidence, protein-truncating variants such as nonsense, canonical splice-site, and frameshift mutations) and moderate-impact (i.e., rare non-synonymous missense variants with deleterious *in silico* predictions [REVEL ≥ 0.64]^33^ or splice-altering predictions [dbscSNV ≥ 0.70 and SpliceAI≥ 0.22] ^34,35^).

For the final screening of variants of interest (comprehensive bioinformatics analysis), we considered the following factors: zygosity-inheritance alignment, gene function, protein expression, description of phenotypes in animals or humans, citation in disease databases, GWAS, and medical literature. Family segregation analysis was performed using Sanger sequencing, depending on DNA availability. Among the genes associated with human phenotypes, pathogenic and likely pathogenic variants were selected based on the ACMG/AMP classification of allelic variant pathogenicity ^36^. Variants of uncertain significance were only included if the associated gene showed a robust correlation with the ACCP phenotype.

### Candidate-gene prioritization

Initially, we identified genes with variants of interest that had previously been associated with pubertal delay or short stature (Figure 1). In contrast, the candidate gene group consisted solely of genes without a known human phenotype listed in the Online Mendelian Inheritance in Man (OMIM) database^37^. Candidate-gene selection was based on several criteria: multiple variant occurrences; GWAS associations with phenotypes such as puberty, height, weight, or other sexual development characteristics; biological plausibility, and gene intolerance to mutation. Gene intolerance was assessed using predictive scores that estimate susceptibility to genetic defects. Assuming that variants in intolerant genes are more likely to be deleterious, we applied the following thresholds: a Loss-of-Function Observed/Expected Upper Fraction (LOEUF) ≤ 0.6 for high-impact variants and a Missense Z-score ≥ 3 for moderate-impact variants^30^.

### Exploring other SLDP cohorts and burden testing

To strengthen the association evidence and refine gene selection, we searched for rare high-impact variants in candidate genes in the multiethnic DPGen Consortium cohort. Subsequently, we conducted a burden test comparing the frequency of rare high-impact variants in candidate genes between SLDP cases and gnomAD v2.0 controls^30^. Case and control data were harmonized using a previously described method^38^, which calibrated the data based on the occurrence of presumed benign synonymous variants. Sequential quality control filters were applied retaining positions with ≥10 read depth in 90% of samples, quality-by-depth > 2, and heterozygous variants with allele balance between 0.2–0.8. Variant counts were aggregated by candidate gene and compared between cases and controls. This comparison included only individuals with adequately sequenced genomic regions. This analysis applied the Fisher's exact test and a 2x2 contingency table. Associations with p-values < 0.05 were considered significant.

### Leveraging UK Biobank data for phenotype association analysis

The UK Biobank is a publicly accessible clinical and genomic database comprising over 500,000 individuals of European ancestry^39^. An exome-wide association study was conducted using WES data from 222,283 women and 178,625 men in the UK Biobank^40^. The aim of this phase was to identify gene-level associations with age at menarche and age at voice breaking phenotypes. Variants with a MAF of < 0.1% were aggregated for each candidate gene showing significant results in the burden test. Variants were classified based on their predicted functional effects into two categories: (1) high-impact variants, defined as high-confidence, protein-truncating variants, and (2) damaging variants, which included both high-impact variants and nonsynonymous missense variants with deleterious predictions *in silico* (CADD score ≥ 25). Gene-phenotype associations with p-values < 0.05 were considered nominally significant.

### Searching for similarities in functional pathways among candidate genes

The Panther Classification System^41^ (version 19.0), a publicly available tool, enables the evaluation of common cellular pathways in groups of genes of interest. The program analyzes the overrepresentation of gene pathways in the human genome relative to the submitted gene list, using Fisher's exact test. We submitted our list of candidate genes, including those with high and moderate impact variants, to identify shared mechanisms of action that might explain their association with each other and with the pubertal delay phenotype. Associations with p-values < 0.05 were considered significant.

## Results

### Patients

A total of 71 patients met the selection criteria for pubertal delay (Supplemental Figures 1 and 2). Table 1 provides the clinical characteristics of the selected patients. The majority of patients were male [61 (85.9%)], and 66 (92.9%) presented with short stature (height Z-score ≤ -2), while 27 (38%) were underweight (-3 ≤ BMI Z-score < -2) at their first evaluation. All patients experienced the onset of puberty before age 18 years, either spontaneously or after receiving low doses of sex steroids. Of these, 21 (30%) individuals required pubertal hormone induction. A family history of pubertal delay was reported in 39 (55%) patients, and 23 (32.4%) had at least one parent with short stature. Adult height (mean height Z-score -1.7) was shorter than target height (mean height Z-score -0.9; p <0.001) and 20 (28%) patients remained short in adulthood.

### Genetic findings associated with growth and puberty disorders

Rare likely pathogenic or pathogenic variants^36^ in genes previously associated with growth disorders (*ANKRD11, CDK13, DPF2, GDF5, KMT2C*, or *TYMP*) were identified in six out of 71 (8.5%) SLDP cases (Supplemental Table 1). Variants in *ANKRD11, DPF2*, and *GDF5* are associated with short stature, while variants in *CDK13* and *DPF2* are linked to poor overall growth. Additionally, variants in *TYMP* are associated with a thin body habitus, and those in *KMT2C* are linked to decreased height ^37^. Importantly, disorders of puberty have not been previously reported in association with any of these genes. None of these syndromes were clinically suspected during patient follow-up.

Furthermore, seven of the 71 patients (9.9%) carried variants in six genes associated with pubertal disorders (*CHD7, GHSR, IGSF10, LGR4, MC3R*, and *TP63*). Among these, two patients (65 and 71) had distinct variants in the *GHSR* gene (p.S84I and p.V182A). The p.V182A variant, classified as likely pathogenic, has been previously studied *in vitro*, showing reduced protein expression on the cell surface^42^. In the *MC3R* gene, the likely pathogenic p.C250S variant has been linked to loss of function in *in vitro* models^43^. Variants in the *TP63* gene are known to cause cleft lip and palate, ectodermal dysplasia, and ectrodactyly syndrome, often accompanied by midline defects and hypogonadism^44^. However, patient 15, carrying the pathogenic p.L335P variant in *TP63*, exhibited only pubertal delay without other features of the syndrome (Supplemental Table 2). The variants identified in *CHD7, GHSR, IGSF10*, and *LGR4* were classified as of uncertain significance.

### Candidate-gene identification

A total of 19 genes were identified as candidates for the SLDP phenotype based on our established criteria for candidate gene selection (Tables 2 and 3). Among the selected high-impact variants—defined as rare, high-confidence, protein-truncating variants—we identified 14 variants in constrained genes (*GPS1, CDK16, NASP, PRDM2, MAP3K4, TRERF1, ARID3B, MYO18A, ATRN, DROSHA, INHBB, FBNP1, SP3*, and *NAMPT*) carried by 12 patients (Table 2). None of these genes had prior descriptions linked to human phenotypes. Additionally, none of these patients carried genetic defects in genes previously associated with pubertal or growth disorders. Among the 14 candidate genes harboring high-impact variants, nine were previously associated with animal phenotypes related to the reproductive system or body size. Furthermore, *INHBB* and *PRDM2* showed signals in GWAS associated with age at menarche and the onset of puberty in boys.

The second group of variants, categorized as moderate impact, included three missense variants predicted to significantly alter splicing (*GTF2A1, MYBL2*, and *CUL5*) and three missense variants (*MYO18A, ACVR2A*, and *GRM4*) found in genes intolerant to amino acid changes (Table 3). Among the six candidate genes harboring moderate-impact variants, two (*GTF2A1* and *ACVR2A*) had been previously linked to reproductive system phenotypes in animal models. Moreover, the *MYO18A* gene was recurrent in the cohort, with a splicing variant identified in patient 44 and the same missense variant observed in patients 2 and 60.

### Burden test results on candidate genes

We extended our search for rare high-impact variants within the 19 candidate genes to the remaining 179 patients in the DPGen Consortium. No additional patients were found to carry high-impact variants in the genes of interest. Subsequently, we conducted a candidate gene burden test on all 250 cases from the DPGen Consortium, which included 113 cases from the USA, 71 from Brazil, 32 from France, 26 from Argentina, and 8 from England. The frequency of rare high-impact variants in the candidate genes from the DPGen Consortium was compared with that observed in a median of 51,874 controls from the gnomAD v2.0 exome database, ensuring adequate sequencing coverage for each candidate gene. Eight genes (*GPS1, INHBB, SP3, NAMPT, ARID3B, NASP, FNBP1*, and *PRDM2*) demonstrated nominal associations with SLDP cases compared to matched controls, with the following p-values: 0.0047, 0.0091, 0.0099, 0.0184, 0.0199, 0.0278, 0.0337, and 0.0486, respectively (Supplemental Table 3).

### Candidate Gene-Phenotype Associations in the UK Biobank

To explore these associations further, we analyzed gene-phenotype relationships, such as age at menarche in 222,283 women and age at voice breaking in 178,625 men, using data from the UK Biobank study. Among the eight genes studied (*GPS1, INHBB, SP3, NAMPT, ARID3B, NASP, FNBP1*, and *PRDM2*), only *INHBB* showed a nominal association with later menarche. Rare high-impact variants in *INHBB* (n = 5 carriers) were linked to an average delay of 1.8 years (p = 0.01).

### Assessing common pathways of action

Supplemental Table 4 shows the pathways enriched for our candidate SLDP genes. Interestingly, there were multiple genes associated with pathways classically implicated in the reproductive system and pubertal timing: the transforming growth factor beta (TGF-β) signaling pathway (*ACVR2, MYO18A* and *INHBB*) and the gonadotropin-releasing hormone (GnRH) receptor pathway (*MAP3K4, ACVR2*, and *INHBB*).

## Discussion

Genetic factors significantly influence pubertal timing, however the genetic basis of SLDP remains poorly understood^42,45,46^. To address this gap, we studied 71 patients with SLDP using WES to identify novel genetic contributors. Six patients had variants in genes linked to syndromes causing short stature, and seven had variants associated with pubertal delay. We identified 14 high-impact and seven moderate-impact variants across 19 candidate genes. Gene burden testing revealed a statistical association between eight candidate genes with high-impact variants and SLDP.

In our cohort, 93% (66/71) of patients presented with short stature at initial evaluation, and 65% (23/66) achieved catch-up growth in adulthood, reaching heights near their genetic target (Table 1). Those who remained short in adulthood were within the confidence intervals of their familial target heights, though short stature prevalence remained higher than anticipated^47^. Adult height among patients with candidate gene variants was not significantly different from the overall cohort (data not shown). These findings collectively suggest familial short stature as a predominant feature in our cohort, independent of puberty timing or genetic background. Previous studies on SLDP have similarly identified familial short stature as predictive of lower adult heigh^47,48^. Additionally, since height and puberty onset are normally distributed traits, their overlap may result from distinct factors. For example, patient 26 had variants in *CDK13* (linked to short stature) and *LGR4* (associated with pubertal delay)^13,49^.

Among the patients with positive genetic findings, six presented with deleterious variants in genes previously associated exclusively with the short stature phenotype ^49–53^. Remarkably, the *ANKRD11* and *CDK13* genes are involved in neuronal development^49,50^, *KMT2C* plays a role in the epigenetic regulation of various genes^51^, *DPF2* is part of the chromatin remodeling complex^52^, and the *TYMP* gene is linked to a significant thinness phenotype^53^. We speculate that the variants found in these genes may have indirectly influenced pubertal development through mechanisms such as altered methylation, regulation of neuronal development, or modulation of energy metabolism.

Mutations in genes associated with isolated hypogonadotropic hypogonadism, such as *GNRHR, TAC*, and *TAC3R*, have been previously described in patients with SLDP^19^. However, none of the patients in our cohort presented with pathogenic variants in these classical hypogonadism-related genes. Conversely, investigating defects in genes related to energy metabolism in patients with delayed puberty is reasonable, given that somatic energy stores play a critical role in regulating behavior, growth, and development^54^. Two children in our cohort carried previously described variants in the *GHSR* gene^42^, which encodes ghrelin, a hormone involved in hunger and satiety regulation. Additionally, a recent study that identified variants in the *MC3R* gene in relation to pubertal delay included a patient from our cohort^43^.

The identification of only a few genes linked to SLDP in a monogenic model suggests that oligogenic or polygenic mechanisms may play a stronger role in its inheritance. In our cohort, more than one variant of interest was identified in six patients with defects in candidate genes. The second variant was either from another candidate gene (n = 2) or from genes previously associated with delayed puberty (n = 4). Notably, oligogenic inheritance has also been linked to hypogonadotropic hypogonadism^17^. The role of polygenicity in puberty onset timing has been highlighted in prior research by the DPGen Consortium, which conducted a polygenic risk score in individuals with SLDP. This study demonstrated that common genetic variants influencing puberty timing in the general population significantly contribute to the genetics of SLDP^55^. Additionally, a multi-ancestry genetic analysis of approximately 800,000 women revealed that girls with very late menarche carried a substantial excess of common alleles^40^.

Burden testing provides population-level evidence for associations between genes and phenotypes. In our analysis, eight genes (*GPS1, INHBB, SP3, NAMPT, ARID3B, NASP, FNBP1*, and *PRDM2*) were significantly enriched in cases of pubertal delay compared to controls. However, the number of individuals with defects in these genes was insufficient to establish a definitive association, achieving only nominal significance (p < 0.05). The high intolerance of these genes to disruptive defects, combined with the rarity of the variants found, made it challenging to identify additional patients, even within a relatively large cohort.

*INHBB* stands out among the candidate genes, as it has also been associated with later age at menarche in UK Biobank participants. This gene encodes the beta B subunit of inhibin, a protein involved in two classes of hormones related to reproductive development: inhibins and activins. These hormones regulate several functions critical to sexual development, including the secretion of hypothalamic and pituitary hormones, gonadal hormone production, and the development and maturation of germ cells^56^. Notably, other candidate genes such as *MAP3K4, MYO18A* and *ACVR2* may be linked to *INHBB*, as they are involved in common signaling pathways, such as the TGF-β pathway and the GnRH receptor pathway. The TGF-β pathway mediates both embryonic and adult signaling functions, providing tissue-specific control of differentiation, proliferation, and cell motility^56^. The GnRH receptor pathway regulates GnRH secretion from the hypothalamus, its action on receptors in the anterior pituitary, and the production and release of gonadotropins^1^.

Our study has limitations that may have influenced the results. The small cohort size considering the low population frequency of the selected variants, phenotypic heterogeneity, and loss of follow-up for most patients limited the power to detect associations and classify variant pathogenicity. The use of gnomAD as a control population introduced potential biases; however, these were minimized through careful harmonization of the data with respect to coverage and quality^57^. Additionally, the lack of functional validation in animal models further restricted the ability to confirm the pathogenicity of identified variants. Larger, more diverse studies are needed to validate these findings.

In conclusion, this study identified 19 candidate genes in a cohort of patients with SLDP. The absence of variants in genes typically associated with isolated hypogonadotropic hypogonadism and the presence of pathogenic variants in genes linked to syndromic forms of short stature might be attributed to the characteristics of our cohort, which predominantly included patients with short stature and SLDP. The candidate genes identified may account for short stature, SLDP, or both conditions. Among the candidate genes, *INHBB* stood out due to its significant association in both the burden test and gene-phenotype correlation, supported by strong biological plausibility. At this stage, the potential to enhance our understanding of the mechanisms governing the onset of puberty, a critical phase of human development, underscores the need for further research in this area.

## Supplementary Material

Fig S1

Fig S2

Supplementary Tables and Figure Legends

## Figures and Tables

**Table 1:** Clinical characteristics of a cohort of individuals with SLDP

	*Total* *N = 71*	*Girls* *N = 10*	*Boys* *N = 61*
*1st evaluation*			
Age*	14.3 (12.8; 15.1)	12.4 (10.9; 12.6)	14.5 (13.5; 15.3)
Height Z-score*	-2.7 (-3.0; -2.3)	-2.6 (-2.8; -2.5)	-2.7 (-3.0; -2.2)
BMI SD*	-1.6 (-2.8; -0.9)	-1.5 (-2.3; -1.0)	-1.7 (-2.8; -0.8)
BA – CA*	-2.9 (-3.6; -2.3)	-2.2 (-2.4; -2.0)	-3.1 (-3.7; -2.4)
*Family history*			
Familial pubertal delay	39 (55%)	3 (30%)	36 (59%)
Familial short stature	23 (32%)	1 (10%)	22 (36%)
Target height Z-score*	-0.9 (-1.5; -0.4)	-0.9 (-1.3; -0.6)	-1.0 (-1.5; -0.4)
*Treatment*			
Pubertal induction	21 (30%)	0	21 (34%)
rhGH	13 (18%)	1 (10%)	12 (20%)
*At puberty*			
Age*	14.6 (13.9; 15.4)	13.1 (12.9; 13.3)	14.8 (14.3; 15.5)
Height Z-score*	-2.8 (-3.1; -2.5)	-2.7 (-3.3; -2.6)	-2.8 (-3.1; -2.4)
BMI Z-score*	-1.8 (-2.8; -1.2)	-1.9 (-2.4; -1.6)	-1.8 (-2.9; -0.9)
*Last evaluation*			
Age*	18.6 (18; 20.4)	17.5 (15.8; 21.2)	18.9 (18.1; 20.4)
Adult height Z-score*	-1.4 (-2.0; -1.0)	-1.1 (-1.6; -0.8)	-1.5 (-2.0; -1.1)

N: number of participants; BMI: body mass index; BA: bone age; CA: chronological age; rhGH: recombinant human growth hormone

* Median and Interquartile range.

**Table 2:** Description of high-impact variants in candidate genes in individuals with SLDP

*ID*	*Gene*	*Coding change*	*Protein change*	*Zygosity*	*MAF*	*LOEUF*	*Animal phenotype*	*GWAS signals*
*13*	*GPS1*	c.1201dupA	p.L400fs	het	0	0.3		
*15*	*CDK16*	c.1240C>T	p.R414*	hem	0	0.6	Male infertility Abnormal testis morphology	
*16*	*NASP*	c.478dupA	p.T160fs	het	9.0e-06	0.4	Embryonic growth arrest Decreased embryo size	Body height
*27*	*PRDM2*	c.4941dupG	p.P1648fs	het from mother	9.0e-06	0.3		Body height Puberty onset measurement Age at menarche Body mass index
*28*	*MAP3K4*	c.634C>T	p.Q212*	het	0	0.4	Decreased body size Primary sex reversal Abnormal testis development	Body height
*40*	*TRERF1*	c.2207delC	p.P736fs	het	0	0.3		Body height
*43*	*ARID3B*	c.1056delT	p.A353fs	het	0	0.3	Embryonic growth retardation Abnormal head morphology	Body height
*44*	*MYO18A*	c.4231-2A>G		het *de novo*	0	0.4	Abnormal embryo size	Body mass index Body height
*57*	*ATRN*	c.1632-2A>G		het	0	0.4	Postnatal growth retardation	
*63*	*DROSHA*	c.859C>T	p.R287*	het	1.7e-04	0.4	Decreased body size	Body height
*63*	*INHBB*	c.874C>T	p.R292*	het	0	0.6	Reduced female fertility	Body height Puberty onset measurement
*63*	*FNBP1*	c.514-1_514insA		het	5.0e-04	0.5		
*66*	*SP3*	c.157-3_157del		het	9.4e-05	0.2	Decreased body size	Body height
*71*	*NAMPT*	c.1348delC	p.E451fs	het	0	0.3		

D: patient identification; MAF: minor allele frequency; LOEUF: Loss of Function Observed/Expected Upper Fraction; GWAS: genome-wide association study; het: heterozygous.

**Table 3:** Description of moderate impact variants in candidate genes in individuals with SLDP

*ID*	*Gene*	*Coding change*	*Protein change*	*Zygosity*	*MAF*	*Z*	*REVEL*	*dbscSNV*	*SpliceAI*	*Animal phenotype*	*GWAS*
*2*	*MYO18A*	c. 4051C>T	p.R1351C	het	1.6e-03	3.5	0.8	0	0	Abnormal embryo size	Body mass index Body height
*16*	*GTF2A1*	c.337G>A	p.A113T	het	1.6e-04	1.2	0.1	1.0	0.7	Decreased testis weight Arrest of spermiogenesis Male infertility	
*21*	*ACVR2A*	c.144A>T	p.E48D	het	8.9e-06	4.7	0.8	0	0	Decreased testis weight Azoospermia Abnormal ovary morphology Delayed male fertility Female infertility	Sex hormone-binding globulin measurement Body height
*30*	*GRM4*	c.2201C>T	p.T734I	het	0	3.9	0.8	0	0		Body height Body mass index
*33*	*MYBL2*	c.500G>A	p.R167K	het	0	2.2	0.6	0.9	0.2		Body height
*60*	*MYO18A*	c. 4051C>T	p.R1351C	het	1.6e-03	3.5	0.8	0	0	Abnormal embryo size	Body mass index Body height
*68*	*CUL5*	c.236G>A	p.R79Q	het from father	1e-04	3.6	0.3	0.8	0.3	Embryonic growth retardation Abnormal embryo size	

ID: patient identification; MAF: minor allele frequency; GWAS: genome-wide association study; het: heterozygous

## Data Availability

The data supporting the findings of this study are not publicly available due to concerns about the privacy of research participants but can be obtained from the corresponding author upon reasonable request.

## Significance Statement

This study investigates the genetic underpinnings of self-limited delayed puberty (SLDP), the most common cause of delayed puberty. By analyzing whole-exome sequencing data from 71 children with SLDP, we identified 21 rare high- and moderate-impact variants in 19 mutation-intolerant candidate genes. Eight of these genes were enriched in cases of SLDP compared to controls in a Burden Test analysis. Notably, *INHBB* was linked to delayed menarche in UK Biobank, suggesting a potential role in pubertal timing pathways. These findings expand the list of genes potentially involved in SLDP, emphasizing its genetic complexity. This research provides new insights into the genetic basis of SLDP, paving the way for improved understanding and management of pubertal disorders and their broader implications in endocrine health.
