# Supplementary figures and images for: Deleterious Variants in Intolerant Genes Reveal New Candidates for Self-Limited Delayed Puberty
Source: Eur J Endocrinol. Author manuscript; Available in PMC 2026 Feb 1. (PMC12013340; doi:10.1093/ejendo/lvaf061)

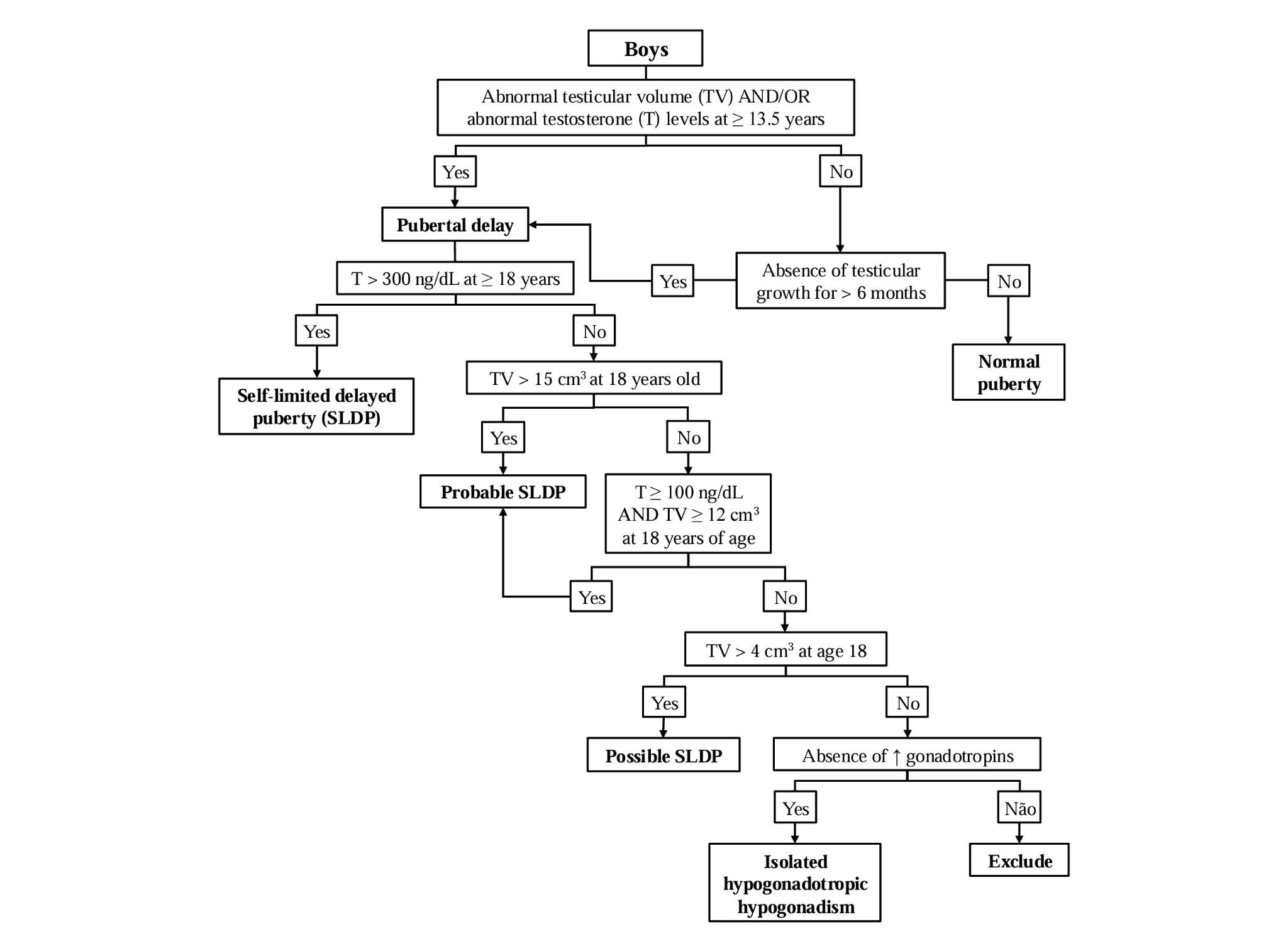

Supplement: Fig S1 [file EMS211999-supplement-Fig_S1.jpeg]

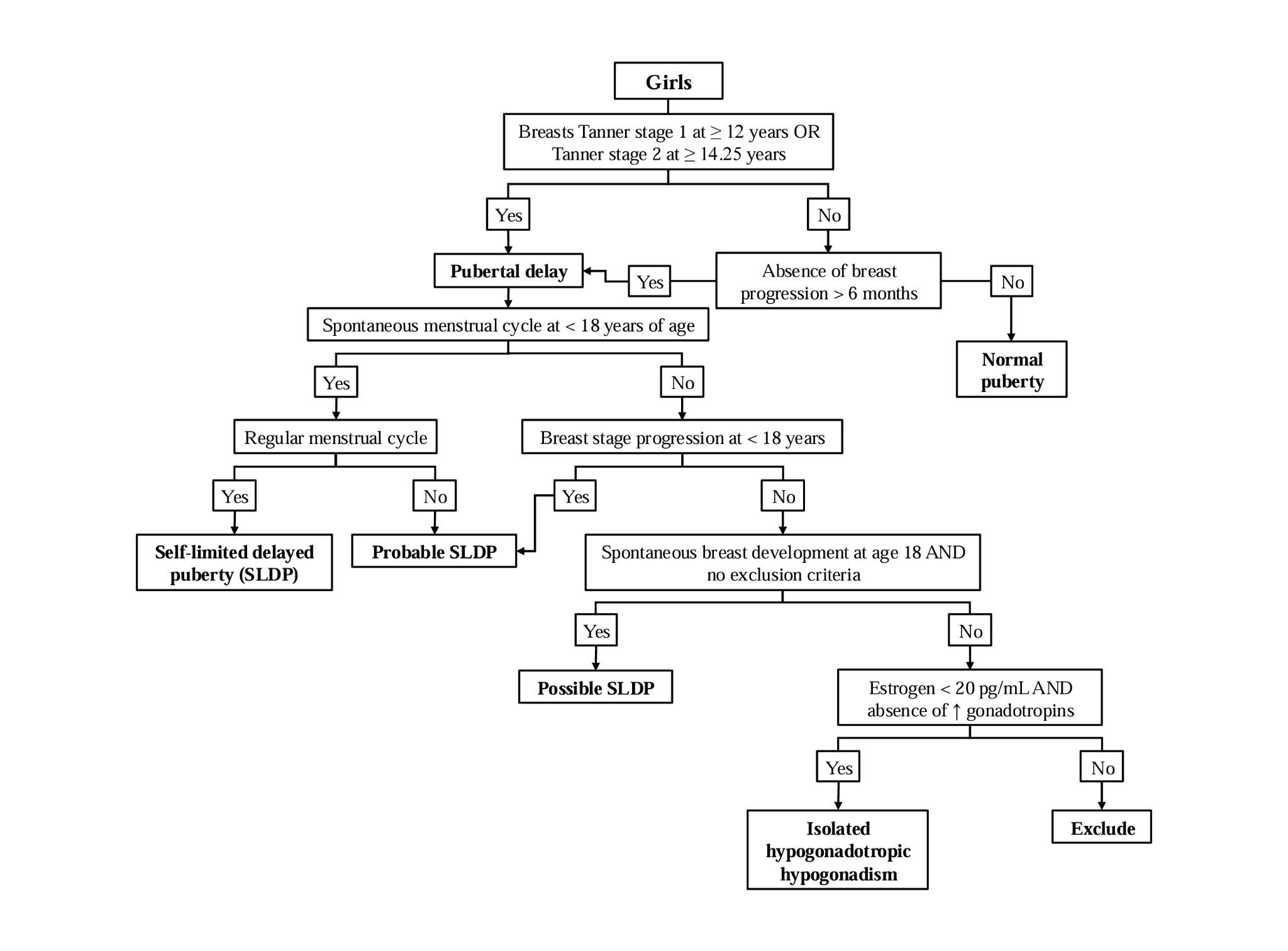

Supplement: Fig S2 [file EMS211999-supplement-Fig_S2.jpeg]
