# Supplementary material for: Deleterious Variants in Intolerant Genes Reveal New Candidates for Self-Limited Delayed Puberty
Source: Eur J Endocrinol. Author manuscript; Available in PMC 2026 Feb 1. (PMC12013340; doi:10.1093/ejendo/lvaf061)
Supplement: Supplementary Tables and Figure Legends [file EMS211999-supplement-Supplementary_Tables_and_Figure_Legends.docx]

**Supplemental File from: Deleterious Variants in Intolerant Genes Reveal New Candidates for Self-Limited Delayed Puberty**

Supplemental Figure 1- Criteria for defining SLDP in boys according to probability levels.

Supplemental Figure 2- Criteria for defining SLDP in girls according to probability levels.

*Supplemental Table 1 – Description of variants previously associated with short stature in individuals with SLDP*

| *ID* | *Gene* | *Coding*  *change* | *Protein change* | *Zygosity* | *MAF* | *#MIM* | *MIM Phenotype-Gene Relationships* | *Inheritance* | *ACMG criteria* |
| --- | --- | --- | --- | --- | --- | --- | --- | --- | --- |
| *10* | *TYMP* | c.1310G>A | p.W437* | hom | 0 | 603041 | Mitochondrial DNA depletion syndrome 1 | AR | LP (PVS1, PM2, PP5) |
| *25* | *DPF2* | c.990C>G | p.C330W | het | 0 | 618027 | Coffin-Siris syndrome 7 | AD | LP (PM2, PP3, PP5) |
| *26* | *CDK13* | c.477dupG | p.L159fs | het de novo | 0 | 617360 | Congenital heart defects, dysmorphic facial features, and intellectual developmental disorder | AD | P (PVS1, PM2, PM6) |
| *42* | *GDF5* | c. 1199G>A | p.C400Y | het | 0 | 112600 | Brachydactyly, type A2 | AD | P (PS4, PM2, PP3, PM1) |
| *64* | *ANKRD11* | c.7195C>T | p.Q2399* | het de novo | 0 | 148050 | KBG syndrome | AD | P (PVS1, PM2, PM6) |
| *70* | *KMT2C* | c.5124_5125insTTTA | p.V1709fs | het | 0 | 617768 | Kleefstra syndrome 2 | AD | LP (PVS1, PM2) |

ID: patient identification; MAF: minor allele frequency; #MIM: Mendelian inheritance of man number; ACMG: American College of Medical Genetics and Genomics; hom: homozygous; het: heterozygous; AR: autosomal recessive; AD: autosomal dominant; LP: likely pathogenic; P: pathogenic.

*Supplemental Table 2 - Description of variants previously associated with pubertal delay in individuals with SLDP*

| *ID* | *Gene* | *Coding change* | *Protein change* | *Zygosity* | *MAF* | *#MIM* | *MIM Phenotype-Gene Relationships* | *Inheritance* | *ACMG criteria* |
| --- | --- | --- | --- | --- | --- | --- | --- | --- | --- |
| *15* | *TP63* | c.1004T>C | p.L335P | het | 0 | 604292 | Ectrodactyly, ectodermal dysplasia, and cleft lip/palate syndrome 3 | AD | LP (PP3, PM2, PP2) |
| *26* | *LGR4* | c.2372C>A | p.P791Q | het from father | 0.0009 | 619613 | Self-limited delayed puberty | AD | VUS (PM2, PP3) |
| *33* | *CHD7* | c.2830C>T | p.R944C | het | 0.00006 | 214800 | CHARGE syndrome | AD | VUS (PM2, PP3, PP2, BP6) |
| *46* | *MC3R* | c.760T>A | p.C254S | het | 0 | 602025 | Delayed puberty^16^ |  | LP (PM2, PS3, PP3) |
| *52* | *IGSF10* | c.7180G>A | p.G2394S | het | 0.002 |  | Delayed puberty^12^ |  | VUS (PM2) |
| *65* | *GHSR* | c.251G>T | p.S84I | het from father | 0 | 615925 | Isolated partial growth hormone deficiency | AD, AR | VUS (PM2, PP3) |
| *71* | *GHSR* | c.545T>C | p.V182A | het from father | 0.0008 | 615925 | Isolated partial growth hormone deficiency | AD, AR | LP (PS4, PP1, PS3, PM2) |

ID: patient identification; MAF: minor allele frequency; #MIM: Mendelian inheritance of man number; ACMG: American College of Medical Genetics and Genomics; het: heterozygous; AR: autosomal recessive; AD: autosomal dominant; LP: likely pathogenic; VUS: variant of uncertain significance.

*Supplemental Table 3 - Burden test comparing the frequency of variants in candidate genes in SLDP cases and controls*

| *Gene* | *DPGen Consortium Cases* | | *gnomAD v2.0 Controls* | | *p -value* |
| --- | --- | --- | --- | --- | --- |
|  | *N of individuals with reliable genotyping* | *N of carriers* | *N of individuals with reliable genotyping* | *N of carriers* |  |
| *GPS1* | 250 | 1 | 53428 | 0 | 0.0047 |
| *INHBB* | 250 | 1 | 54699 | 1 | 0.0091 |
| *SP3* | 250 | 1 | 49956 | 1 | 0.0099 |
| *NAMPT* | 250 | 1 | 53854 | 3 | 0.0184 |
| *ARID3B* | 250 | 1 | 49614 | 3 | 0.0199 |
| *NASP* | 250 | 1 | 53112 | 5 | 0.0278 |
| *FNBP1* | 250 | 1 | 50980 | 6 | 0.0337 |
| *PRDM2* | 250 | 1 | 50082 | 9 | 0.0486 |
| *ATRN* | 250 | 1 | 54108 | 14 | 0.0668 |
| *DROSHA* | 250 | 1 | 53326 | 14 | 0.0678 |
| *MAP3K4* | 250 | 1 | 42590 | 11 | 0.0678 |
| *MYO18A* | 250 | 1 | 52544 | 42 | 0.1847 |
| *GTF2A1* | 250 | 0 | 52414 | 2 | 1 |
| *ACVR2A* | 250 | 0 | 54092 | 1 | 1 |
| *GRM4* | 250 | 0 | 54295 | 14 | 1 |
| *MYBL2* | 250 | 0 | 52729 | 7 | 1 |
| *CUL5* | 250 | 0 | 50029 | 2 | 1 |

N: number; DPGen: Delayed puberty genetics.

*Supplemental Table 4 - Enrichment of cellular pathways in candidate genes*

| *PANTHER Pathways* | *Homo sapiens* | *Candidate genes* | | | |
| --- | --- | --- | --- | --- | --- |
|  | *N* | *N* | [*expected*](https://pantherdb.org/tools/compareToRefList.jsp?sortOrder=2&sortList=Client%20Text%20Box%20Input&sortField=exp) | *Fold Enrichment* | *p value* |
| General transcription regulation | 39 | 1 | 0.04 | 27.8 | 0.04 |
| p38 MAPK pathway | 41 | 1 | 0.04 | 26.4 | 0.04 |
| TGF-beta signaling pathway | 100 | 3 | 0.09 | 21.7 | 0.004 |
| Oxidative stress response | 54 | 1 | 0.05 | 20.1 | 0.05 |
| Gonadotropin-releasing hormone receptor pathway | 231 | 3 | 0.21 | 14.1 | 0.001 |

N: number of pathways.
